# Vertically-Ordered Mesoporous Silica Films for Electrochemical Detection of Hg(II) Ion in Pharmaceuticals and Soil Samples

**DOI:** 10.3389/fchem.2022.952936

**Published:** 2022-06-29

**Authors:** Mengqi Zhang, Yanqi Zou, Xiaoyu Zhou, Fei Yan, Zhanling Ding

**Affiliations:** ^1^ Guangxi Medical University Cancer Hospital, Nanning, China; ^2^ Department of Chemistry, Key Laboratory of Surface & Interface Science of Polymer Materials of Zhejiang Province, Zhejiang Sci-Tech University, Hangzhou, China

**Keywords:** vertically-ordered mesoporous silica film, mercury ion, electrochemical detection, pharmaceutical, soil

## Abstract

Rapid and simple determination of mercury ion (Hg^2+^) in pharmaceuticals and soil samples is vital for human health and the environmental monitoring. Vertically-ordered mesoporous silica films (VMSF) supported by the indium tin oxide (ITO) electrode surface were prepared by electrochemically assisted self-assembly method and utilized for electrochemical detection of Hg^2+^. Owing to the negatively charged channel walls and ultrasmall pore diameter, VMSF displays obvious cationic selectivity and has highly electrostatic interaction for Hg^2+^, giving rise to the strong electrochemical signals. By recording the anodic stripping signals of adsorbed Hg^2+^ using differential pulse voltammetry, quantitative detection of Hg^2+^ was achieved with a wide linear range (0.2 μM–20 μM) and a low limit of detection (3 nM). Furthermore, considering the anti-fouling and anti-interference capacity of VMSF, the proposed VMSF/ITO sensor has been successfully applied to detect Hg^2+^ in pharmaceuticals and soil samples without tedious pretreatment processes of samples.

## Introduction

Heavy metal ions are one of the most dominating exogenous pollutants in traditional Chinese medicine and also one of the major environmental pollutants affecting the whole world (Li et al., 2019), which have serious effects on human health and the environment (Wang et al., 2010; Ge et al., 2019). As they are difficult to be degraded by microorganism, heavy metal ions not only tend to accumulate in the environment that turn to more toxic methyl compounds (Wang et al., 2020), but also accumulate in the human body through the food chain at harmful concentrations, endangering human health seriously (Ding et al., 2021; Guo et al., 2021). Mercury ion (Hg^2+^) as one of the most dangerous metals in the ecosystem is nondegradable in food, medicine and biological systems, posing a serious threat to public health and environmental balance (Radwan et al., 2020). Due to its high affinity for sulphur of enzymes and proteins, Hg^2+^ can enter into the human body through a variety of pathways and then hinder normal cell metabolism, leading to the muti-system damage (mainly neurotoxic and nephrotoxic) even at very low concentrations (Rullyani et al., 2019; Zhang et al., 2019; Bao et al., 2021). Moreover, when the Hg^2+^ content in soil is excessive, Hg^2+^ will accumulate in plants and produce toxins, and even lead to the death of plants (Wu et al., 2011; Xiong et al., 2017; Feng et al., 2018). Therefore, it’s of great significance for the quantitative monitoring of Hg^2+^ in medicine and environment. Up to now, a number of analytical methods have been applied to the detection of Hg^2+^, for example, colorimetry (Gao et al., 2014; Balasurya et al., 2020), atomic absorption spectrometry (AAS) (Hsu et al., 2011), fluorescence spectrometry (Fang et al., 2018; Mao et al., 2019), gas chromatography (GC) (Miranda-Andrades et al., 2019), etc. However, these traditional analysis methods require large equipment, high cost, complex operation and sample pre-processing. Electrochemical method has received attractive attention due to its rapidity, ease of use, and low cost (Manjunatha et al., 2013; Lu et al., 2018; Hareesha et al., 2019; Hareesha and Manjunatha, 2020a; Hareesha and Manjunatha, 2020b; Charithra et al., 2020).

Recently, porous materials as functional building blocks have been widely used for the construction of high-performance sensors, taking advantage of their ease of hybridization (Cui et al., 2020; Cui et al., 2021; Duan et al., 2021; Liu et al., 2022), efficient enrichment (Liang et al., 2021; Yan et al., 2021), and rapid mass transfer (Zhou et al., 2022a; Gong et al., 2022). Especially, vertically-ordered mesoporous silica films (VMSF) have been proven to be powerful preconcentration materials for electrochemical detection of metal ions, such as Cu^2+^ (Cheng et al., 2018; Lu et al., 2018), Hg^2+^ (Lu et al., 2018), Ag^+^ (Herzog et al., 2013), Pb^2+^ (Fernández et al., 2014; Cheng et al., 2018; Li et al., 2021), and Cd^2+^ (Cheng et al., 2018; Lu et al., 2018; Li et al., 2021). VMSF prepared by electrochemically assisted self-assembly (EASA) (Walcarius et al., 2007) and Stöber solution growth (Teng et al., 2012) methods possess a perpendicularly regular channel structure with uniform pore size, negatively charged channel walls and high porosity, which are very suitable for the construction of electrochemical sensors for direct analysis of various analytes in complex samples, including biomolecules (Li et al., 2014; Ma et al., 2022a; Ma et al., 2022b; Zhu et al., 2022), drug molecules (Wang et al., 2022; Wei et al., 2022), organic pollutants (Yan et al., 2021), metal ions (Cheng et al., 2018; Lu et al., 2018). The excellent electrochemical signals of metal ions obtained at the VMSF modified sensors rely on the electrostatic enrichment between cationic metal ions and negatively charged channel walls of VMSF. Moreover, modification of VMSF with diversified functional materials inside the nanochannels or underlying surface could improve the detection performance, such as organosilanes (Etienne et al., 2009; Herzog et al., 2013), graphene nanosheets (Zhou et al., 2022b), and graphene quantum dots (Lu et al., 2018). Exploitation of such VMSF without further functionalization for electrochemical detection of Hg^2+^ has not been studied, to the best of our knowledge.

In this work, VMSF with pore diameter of 2∼3 nm was prepared on the indium tin oxide (ITO) electrode by EASA method, which was then employed to detect Hg^2+^ using anodic stripping voltammetry. Arising from the ultrasmall channel diameter and negatively charged channel walls, VMSF provides apparent electrostatic attraction to Hg^2+^, ultimately generating excellent electrochemical signals. Such VMSF/ITO sensor is able to detect Hg^2+^ with a wide linear range (0.2 μM–20 μM) and a low limit of detection (3 nM). Furthermore, owing to the anti-fouling and anti-interference capacity of VMSF, the proposed sensor has been successfully applied to detect Hg^2+^ in pharmaceuticals and soil samples without complex sample pretreatments.

## Materials and Methods

### Chemical and Materials

All chemicals of analytical grade were used as received without further purification. Ultrapure water (18.2 MΩ cm, Milli-Q, Millipore) was used to prepare all aqueous solutions. Mercury nitrate monohydrate (Hg (NO_3_)_2_·H_2_O, AR) and sodium phosphate dibasic dodecahydrate (Na_2_HPO_4_·12H_2_O, 99%) were obtained from Macklin (China). Iron chloride (FeCl_3_, 99.9%), sodium phosphate monobasic dihydrate (NaH_2_PO_4_·2H_2_O, 99%), cadmium nitrate tetrahydrate (Cd(NO_3_)_2_·4H_2_O, 99%), cetyltrimethylammonium bromide (CTAB), tetraethoxysilane (TEOS, 98%), hydroxypropylmethylcellulose (HPMC), potassium ferricyanide (K_3_ [Fe(CN)_6_], 99.5%), potassium hydrogen phthalate (KHP, 99.8%), starch soluble (Starch, 99.0%), humic acid (HA, 90%) and lauryl sodium sulfate (SDS, 98.5%) were all purchased from Aladdin (China). Lignin (50%) was received from Solarbio. Sodium nitrate (NaNO_3_) was ordered from Wuxi Zhangwang Chemical Reagent (China). Zinc chloride (ZnCl_2_, 98%) was purchased from YongDa Chemical Reagent (China). Hexaammineruthenium (III) chloride (Ru(NH_3_)_6_Cl_3_, 98%) was received from Sigma (USA). Potassium chloride (KCl, 99.5%), sodium chloride (NaCl, 99.5%), and magnesium chloride (MgCl_2_, 95%), were purchased from Hangzhou Gaojing Fine Chemical Reagent (China). Bezoar antidotal pills were ordered from Ali health pharmacy (China). Soil and pond water were collected from Xiasha Campus of Zhejiang Sci-Tech University (China). Indium tin oxide (ITO) coated glasses (<17 Ω/square, thickness: 100 ± 20 nm) were purchased from Zhuhai Kaivo Optoelectronic Technology (China).

### Measurements and Instrumentations

Transmission electron microscopy (TEM) images were recorded from the HT7700 microscope (JEOL, Japan) operated at 100 kV. The scanning electron microscopy (SEM) image was obtained from the SU8010 (Hitachi, Japan) at an acceleration voltage of 5 kV. Electrochemical measurements including cyclic voltammetry (CV) and differential pulse voltammetry (DPV) were collected from the Autolab (PGSTAT302N) electrochemical workstation (Metrohm, Switzerland). A conventional three-electrode system was adopted, with bare or modified ITO as working electrode, Ag/AgCl as the reference electrode, and platinum electrode as the counter electrode. The DPV parameters were as follows: step, 5 mV; modulation time, 0.05 s; modulation amplitude, 50 mV; interval time, 0.2 s.

### Preparation of Vertically-Ordered Mesoporous Silica Films/Indium Tin Oxide

**SCHEME 1:**
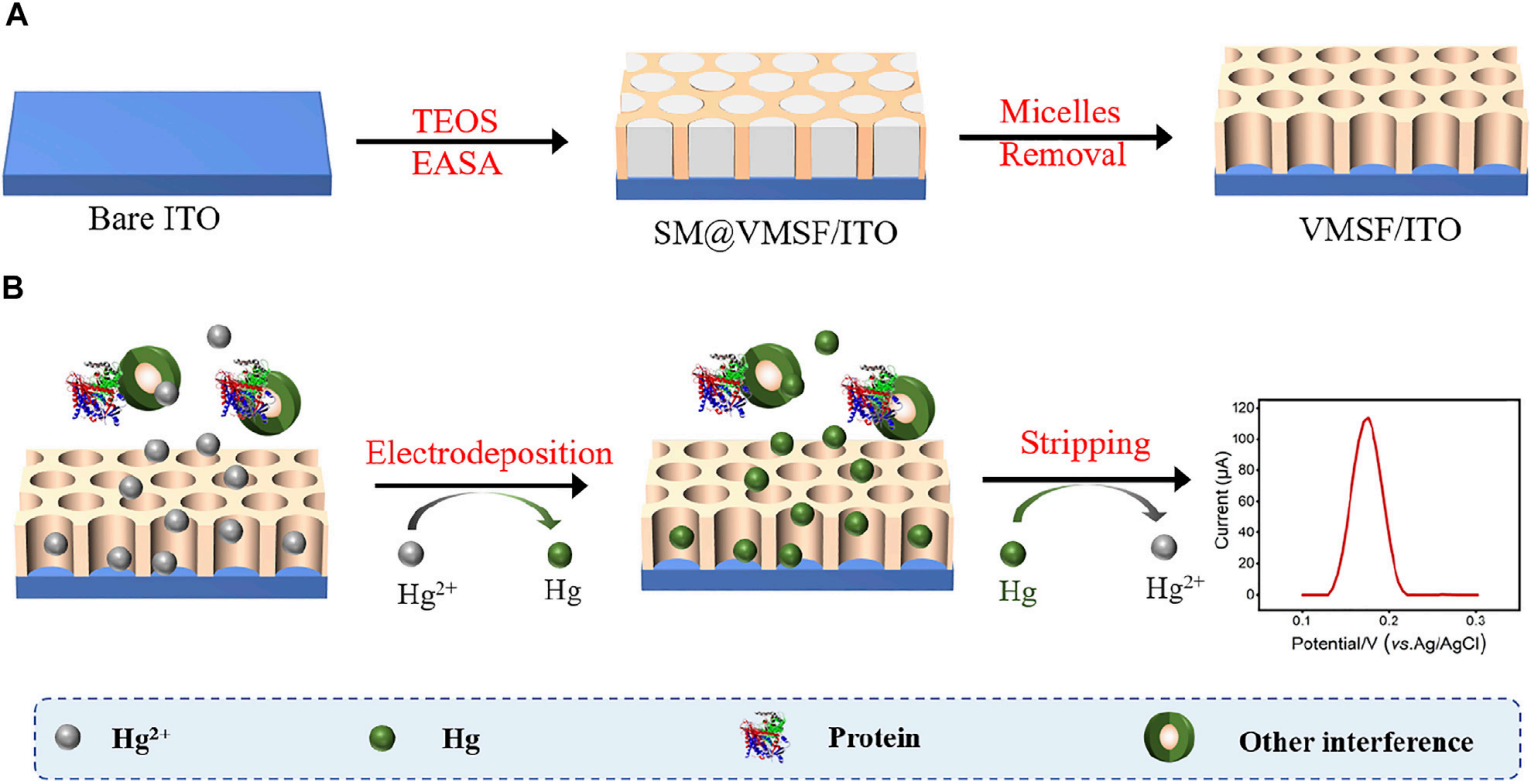
The preparation of VMSF/ITO electrode **(A)** and detection of Hg^2+^ **(B)**.

ITO electrodes require a cleaning process prior to use. Firstly, the ITO electrodes were soaked into 1 M NaOH aqueous solution at the room temperature overnight, and ultrasonicated in acetone, ethanol and deionized water for each 0.5 h sequentially. After being dried by nitrogen stream, freshly cleaned ITO electrodes were obtained. Then, VMSF was grown onto the ITO electrode surface (VMSF/ITO) using the electrochemically assisted self-assembly (EASA) method (Scheme 1.) (Herzog et al., 2013) Briefly, 1.585 g CTAB was first dissolved in a mixture of 20 ml ethanol and 20 ml sodium nitrate (0.1 M, pH 2.6), and then mixed with 3050 μl TEOS. Above mixture stirred for 2.5 h to prepare silica-based precursor. Subsequently, the cleaned ITO electrode was immersed into the precursor and suffered from a constant current density (−1.3 mA/cm^2^) for 10 s. After reaction finished, the electrode was removed from the solution immediately, rinsed with deionized water, dried with nitrogen stream and aged at 120°C for 12 h overnight. The resulting electrode with surfactant micelles (SM) inside the nanochannels of VMSF was designated as SM@VMSF/ITO. Finally, the VMSF/ITO electrode was obtained by solvent extraction of SM with 0.1 M HCl/ethanol solution under stirring for 5 min.

### Detection of Hg^2+^

Hg^2+^ was determined by anodic stripping voltammetry (ASV). Briefly, −0.8 V was applied to the VMSF/ITO electrode (the electrode area is 0.5 × 1 cm^2^) in 0.1 M PBS (pH 5.0) containing Hg^2+^ for 300 s to electrochemical deposition and reduction of Hg^2+^. Then the anodic stripping current of Hg was recorded in 0.1 M PBS (pH 5.0) by DPV method.

### Preparation of Real Samples

The preparation of bezoar antidotal pill sample: 0.500 g pills were placed in a 100 ml beaker and 5 ml nitric acid was added and placed overnight. The sample was slowly digested by heating on the induction cooker, and nitric acid was continuously added until the digests were complete. Then, a certain amount of perchloric acid was added (the dosage ratio of perchloric acid to nitric acid was 1:4), and the solution was heated and evaporated to 1–2 ml. After being cooled to room temperature, above solution was diluted with 0.1 M PBS (pH 5.0) to 50 ml. Soil sample for direct analysis was obtained by dispersing soil with 0.1 M PBS (pH 5.0) to obtain 0.1 mg/ml dispersion.

## Results and Discussion

### Characterization of the Vertically-Ordered Mesoporous Silica Films/Indium Tin Oxide

**FIGURE 1:**
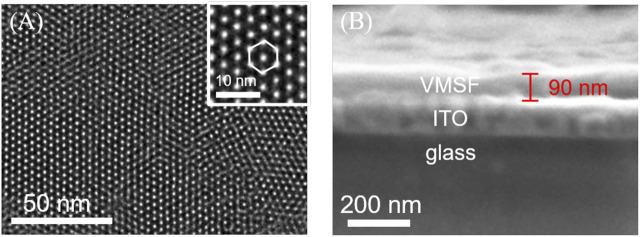
**(A)** Top-view TEM and **(B)** cross-sectional view SEM images of VMSF. The inset in **(A)** is the corresponding magnified image.

**FIGURE 2:**
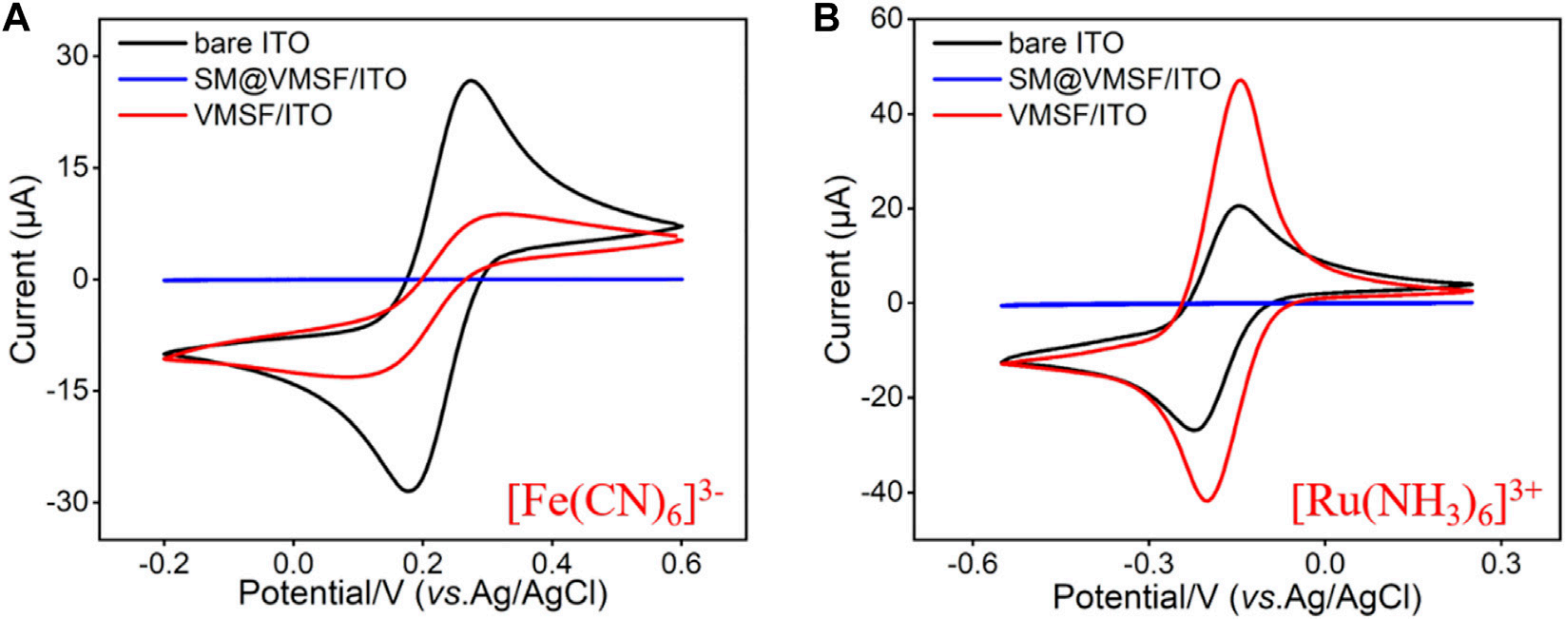
CV curves obtained from the bare ITO, SM@VMSF/ITO and VMSF/ITO electrodes in [Fe(CN)_6_]^3−^ **(A)** and [Ru(NH_3_)_6_]^3+^ **(B)** solution (0.5 mM in 0.05M KHP, adjusted the pH to 7.4). The scan rate was 50 mV/s.

TEM and SEM were employed to characterize the morphology and thickness of the VMSF. Figure 1 shows top-view TEM (A) and cross-sectional view SEM (B) images of the VMSF. It could be found from top-view TEM image that silica mesopores displayed as bright spots are uniformly ordered in a hexagonal shape and their diameter is about 2.4 nm (Figure 1A). And the cross-sectional view SEM image displays apparently three layers from top to bottom, corresponding to VMSF, ITO and glass substrate. The VMSF possesses homogeneous thickness of ∼90 nm (Figure 1B). Then two charged electrochemical probes with opposite charge (Fe(CN)_6_^3−^ and Ru(NH_3_)_6_^3+^) were used to study the intactness and molecular permeability of VMSF using cyclic voltammetry (CV) technique. CV responses of Fe(CN)_6_^3−^ and Ru(NH_3_)_6_^3+^ at bare ITO, SM@VMSF/ITO and VMSF/ITO electrodes were compared in Figure 2 As shown, a pair of obvious redox current peaks was observed for Fe(CN)_6_^3−^ and Ru(NH_3_)_6_^3+^ at the bare ITO electrode (black line). When the impermeable SM@VMSF layer was grown onto the bare ITO surface, the redox current peaks were shielded and only capacitive currents were exhibited (blue line), proving the full coverage of VMSF without cracking on the bare ITO electrode surface. After the removal of SM from the nanochannels of VMSF, the recovered electrochemical signals of Fe(CN)_6_^3−^ and Ru(NH_3_)_6_^3+^ were obtained at the VMSF/ITO electrode. Moreover, the electrochemical signal of Fe(CN)_6_^3−^ was decreased but that of Ru(NH_3_)_6_^3+^ was increased at the VMSF/ITO (red line), compared to that of the bare ITO electrode. Such obvious charge permselectivity is due to the deprotonation of the silanol groups (p*K*_a_ ∼ 2–3) on the channel walls of VMSF in the experimental condition (pH∼7.4). All above results are in accordance with the previous reports (Lin et al., 2015; Zhou et al., 2022c).

### Electrochemical Behavior of Hg^2+^ at the Vertically-Ordered Mesoporous Silica Films/Indium Tin Oxide Electrode

**FIGURE 3:**
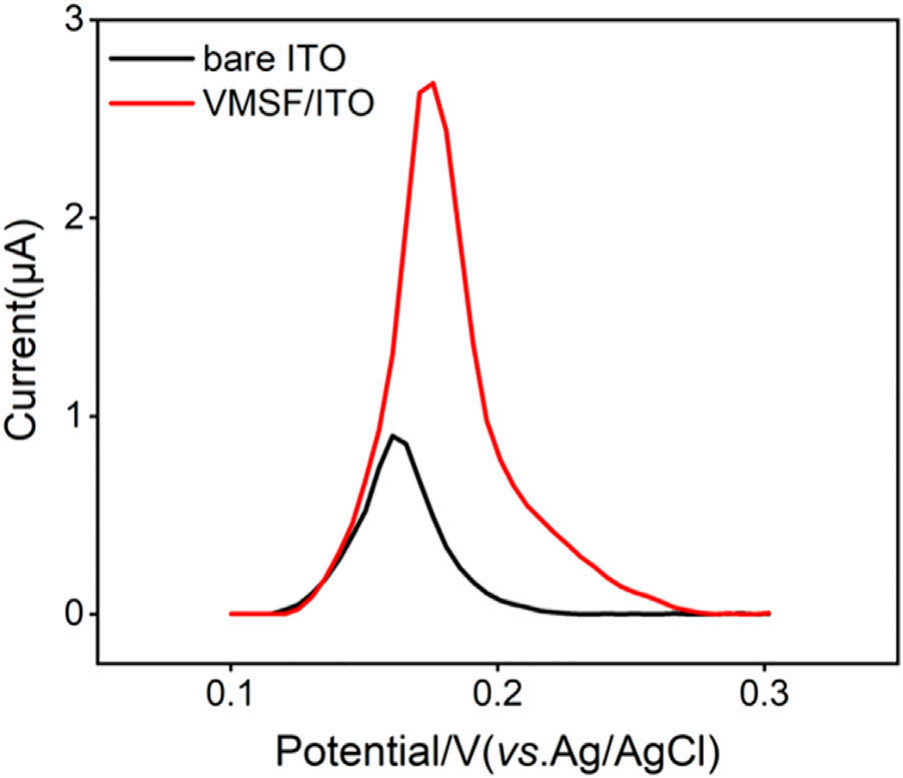
DPV curves obtained from the bare ITO and VMSF/ITO electrodes in response to 1 μM Hg^2+^ in 0.1 M PBS (pH 5.0).

As shown in Scheme 1, Hg^2+^ was determined by VMSF/ITO electrode using anodic stripping voltammetry (ASV), namely the electrochemical deposition and reduction of Hg^2+^ and the following anodic stripping. As illustrated, Hg^2+^ could be enriched in the nanochannels of VMSF through electrostatic effect between cationic Hg^2+^ and VMSF with negative surface charges. Then Hg^2+^ confined in the nanochannels underwent −0.8 V for 300 s and be reduced to elemental mercury. Anodic stripping signals of elemental mercury was collected by differential pulse voltammetry (DPV) in the potential range from 0.1 V to 0.3 V. Figure 3 compares the stripping signals of the bare ITO and VMSF/ITO electrodes in response to the 1 μM Hg^2+^ dissolved in 0.1 M PBS (pH 5.0). Apparently, both bare ITO and VMSF/ITO electrodes gives stripping current peak at 0.16 V, corresponding to the characteristic potential of Hg^2+^. However, the magnitude of the stripping signal of Hg^2+^ at the VMSF/ITO was nearly 3-fold larger than that of bare ITO, confirming the preconcentration capacity of VMSF.

### Optimal Conditions for Hg^2+^ Detection

**FIGURE 4:**
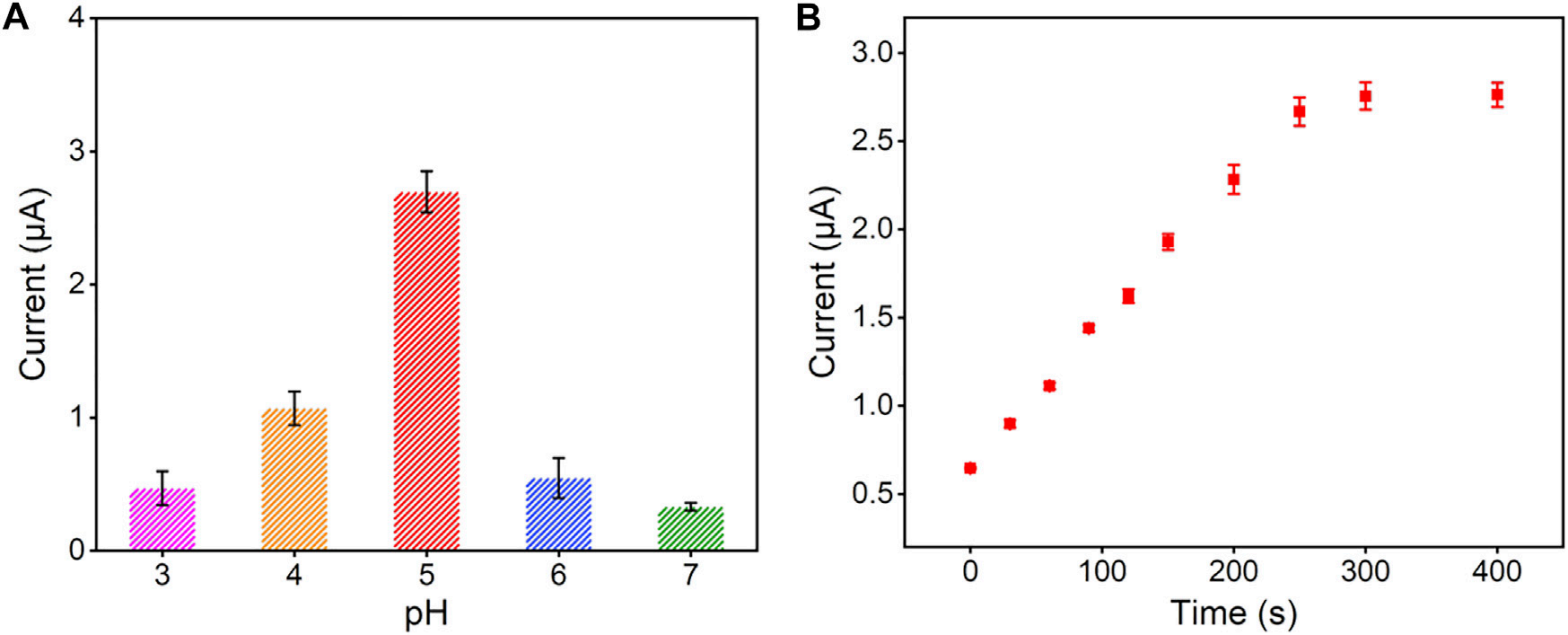
Effect of experimental conditions on the detection performance of Hg^2+^: **(A)** pH of supporting electrolyte and **(B)** preconcentration time. The concentration of Hg^2+^ is 1 μM and the error bars represent the standard deviations (SD) of three measurements.

To obtain a highly sensitive performance for the detection of Hg^2+^, we optimized the detection conditions (pH value and electrodeposition time). With the pH value of supporting electrolyte increased, the stripping signal of Hg^2+^ reached the highest at the pH value of 5.0 (Figure 4A), which was selected for following experiments. This is because that in the high pH range, Hg^2+^ is prone to hydrolysis and in the low pH range, nanochannel walls of VMSF tend to be uncharged. As shown in Figure 4B, the stripping signal increased with the increase of electrodeposition time, and reached a plateau at the time of 300 s. Therefore, 300 s was chosen as the optimal electrodeposition time for Hg^2+^.

### Electrochemical Detection of Hg^2+^ Using Vertically-Ordered Mesoporous Silica Films/Indium Tin Oxide Electrode

**FIGURE 5:**
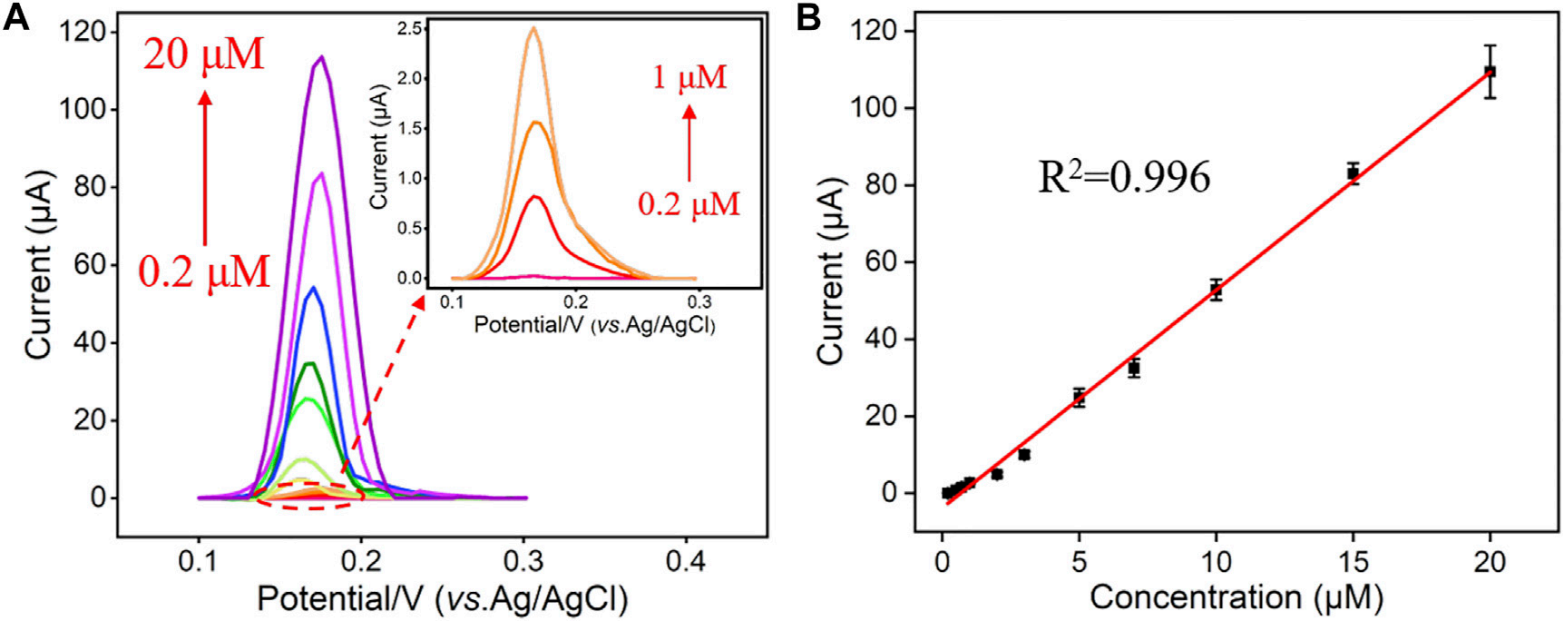
**(A)** DPV curves obtained from the VMSF/ITO in response to different concentrations (0.2 μM, 0.5 μM, 0.7 μM, 1 μM, 2 μM, 3 μM, 5 μM, 7 μM, 10 μM, 15 μM, and 20 μM) of Hg^2+^. **(B)** The dose response curve of Hg^2+^ and the error bars represent the SD of three measurements.

Under the optimal experimental conditions described above, a series of different concentrations of Hg^2+^ were detected by the VMSF/ITO electrode and the results were shown in Figure 5A. As seen, the stripping signal obtained from the VMSF/ITO electrode increased with the increasing concentrations of Hg^2+^ in the range of 0.2 μM–20 μM. A good linear relationship was found between the stripping peak current and Hg^2+^ concentration, yielding a fitting linear regression equation of *I* (μA) = 5.65 *C* (μM)—3.77 (*R* ^2^ = 0.996). And the calculated limit of detection (LOD) for Hg^2+^ was 3 nM (S/N = 3). In comparison with other electrochemical methods for Hg^2+^ detection (Table 1), the VMSF/ITO electrode is not good in terms of linear range and LOD. However, the proposed sensor shows distinct advantages: (1) Owing to the size selectivity of the VMSF, biomacromolecules such as protein cannot reach the underlying ITO surface and passivate electrode, which makes the as-prepared VMSF/ITO sensor excellent anti-fouling property. (2) VMSF with charge permselectivity could avoid the interferences of electronegative molecule, leading to the decreased background signal. (3) The electrode is more cost-effective than other modified electrodes with nanomaterials and the preparation process is rather simple.

**TABLE 1:** Comparison of the analytical performances of various electroanalytical methods for the determination of Hg^2+^.

Electrodes	Method	Linear range (μM)	LOD (nM)	Ref.
IIP-CILE	DPV	0.0005–0.01	0.1	Bahrami et al. (2015)
		0.08–2		
Ag-rGO/GCE	DPV	0.000037–0.367	0.018	Ma et al. (2021)
Au@HS-rGO	DPV	0.001–0.2	0.38	Zhao et al. (2019)
Au-DMAET-(SWCNT-PABS)	SWASV	0.07–0.9	63.4	Matlou et al. (2016)
ZnO-NP/CPE	SWV	3–21	0.43	Moutcine et al. (2020)
Ru/CeO_2_/GCE	SWASV	0.06–0.8	19	Sun et al. (2020)
VMSF/ITO	SWASV	0.2–20	3	This work

IIP-CILE, ion imprinted polymeric nanobeads-carbon ionic liquid paste electrode; rGO, reduced graphene oxide; GCE, glassy carbon electrodes; DMAET, dimethyl amino ethane thiol; SWCNT-PABS, single walled carbon nanotube-poly (m-amino benzene sulfonic acid); NP, natural phosphate; CPE, carbon paste electrode.

### Anti-interference and Anti-fouling of the Vertically-Ordered Mesoporous Silica Films/Indium Tin Oxide Electrode

**FIGURE 6:**
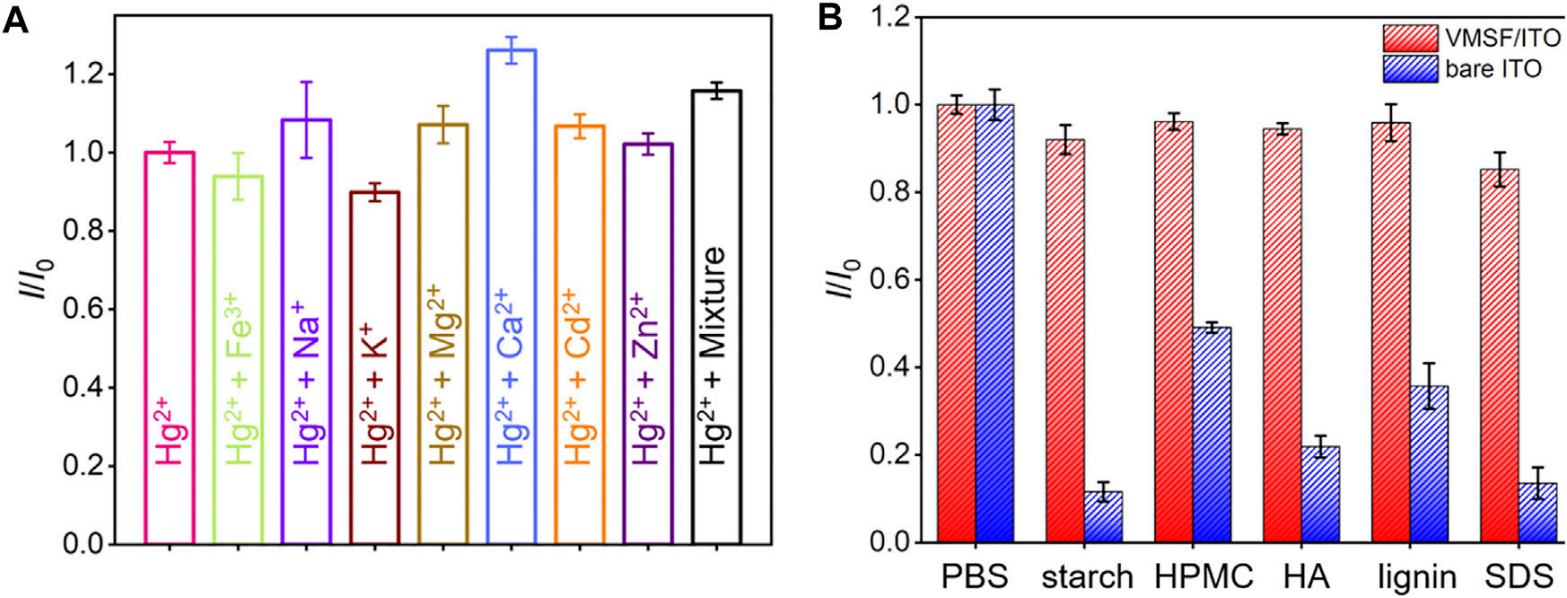
**(A)** The current ratio (*I*/*I*_0_) obtained from the VMSF/ITO electrode for the detection of 1 μM Hg^2+^ in the absence (*I*_0_) and presence (*I*) of 100 μM different metal ions. **(B)** Comparison of the *I*/*I*_0_ obtained from the VMSF/ITO and bare ITO electrode for the detection of 1 μM Hg^2+^ in the absence (*I*_0_) and presence (*I*) of complex interfering species (50 μg/ml starch, HPMC, HA, Lignin and SDS). The error bars represent the SD of three measurements.

A series of metal ions (Fe^3+^, Na^+^, K^+^, Mg^2+^, Cd^2+^, and Zn^2+^) potentially existed in complex samples were selected as interfering substances to investigate the anti-interference ability of VMSF/ITO sensor. As shown in Figure 6A, these metal ions have no obvious interference on the Hg^2+^ detection, which is attributed to the characteristic potential of Hg^2+^. Moreover, anti-fouling performance of VMSF/ITO in complex matrix was also studied using fouling species (starch, HPMC, HA, lignin, and SDS). Compared to the bare ITO electrode, stripping signals of the VMSF/ITO electrode remain unchanged (Figure 6B), showing the good anti-fouling capacity of VMSF. Above results indicate that the proposed VMSF/ITO sensor possesses excellent anti-fouling and anti-interference ability and has great potential in direct electrochemical analysis of real samples. However, the proposed VMSF/ITO electrode has not long-term stability in strong alkaline solutions due to the hydrolysis of VMSF.

### Hg^2+^ Analyses in Pharmaceuticals and Soil Samples

**FIGURE 7:**
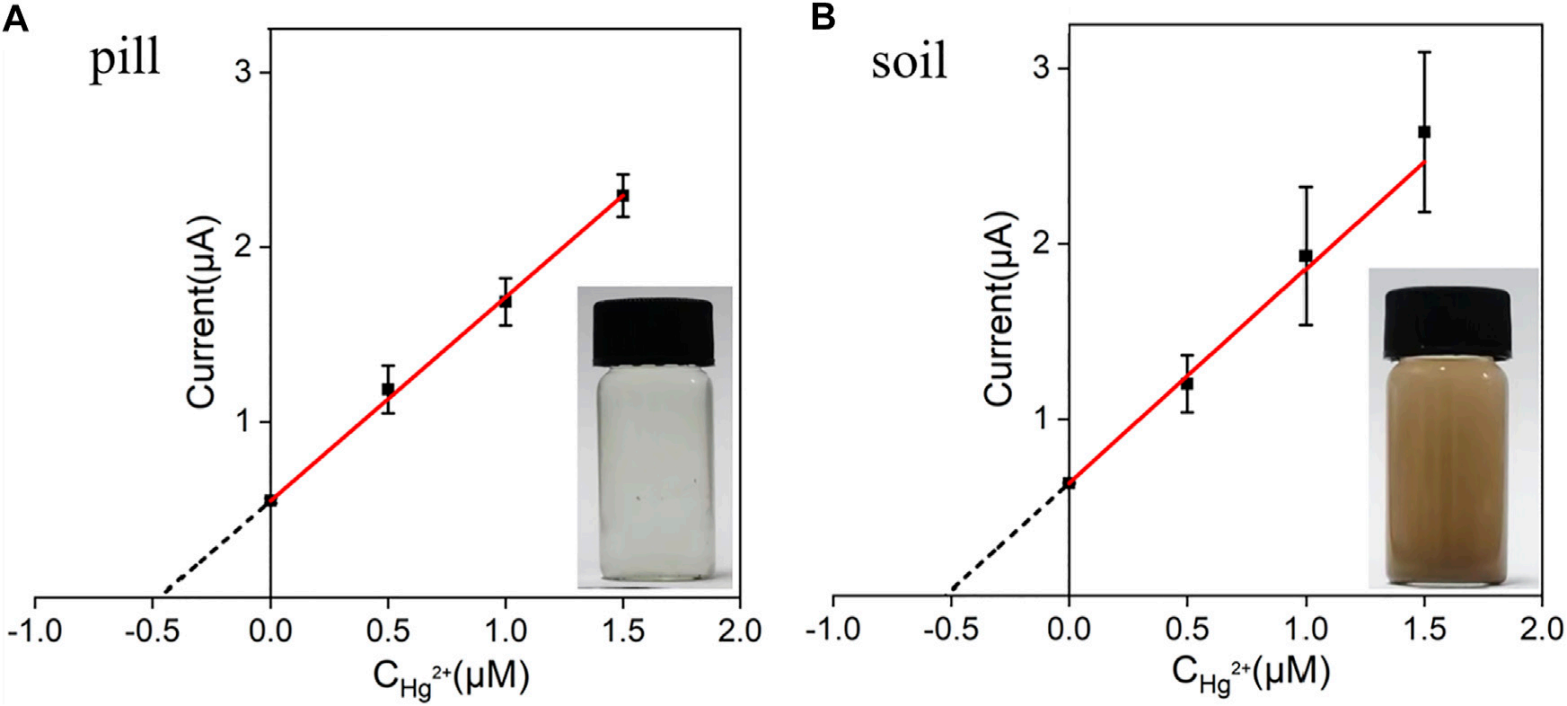
The linear relationship between the anodic stripping currents and Hg^2+^ concentration. Anodic stripping currents were measured by the VMSF/ITO electrode in bezoar antidotal pill **(A)** and soil **(B)** samples before and after the addition of a certain amount of Hg^2+^. The error bars represent the SD of three measurements.

Bezoar antidotal pill and soil samples for direct analysis were obtained by the artificial addition of 0.50 μM Hg^2+^. Afterwards, the concentrations of Hg^2+^ presented in these two samples were determined by standard addition method using VMSF/ITO electrode and the results were shown in Figure 7. As seen, the concentrations of Hg^2+^ in bezoar antidotal pill and soil samples were measured to be 0.48 μM and 0.52 μM through extrapolation of the linear line to the concentration axis, respectively, which were very close to the theoretical value (0.50 μM), indicating that our sensor have a good application prospect in the detection of pharmaceuticals and environmental samples.

## Conclusion

In summary, ITO electrodes modified with VMSF were prepared and used as an electrochemical sensor for the detection of Hg^2+^ by using anodic stripping voltammetry. VMSF with ultrasmall pore size, negatively charged channel walls and large internal surface displays apparent electrostatic attraction to Hg^2+^, ultimately producing excellent electrochemical signals. A wide linear range (0.2 μM–20 μM) and a low limit of detection (3 nM) were obtained at the VMSF/ITO sensor. And the excellent anti-fouling and anti-interference ability of VMSF allow the quantitative determination of Hg^2+^ in pharmaceutical and soil samples without tedious sample pretreatments. Such VMSF perpendicularly tethered to the electrode can be prepared by simple operation without any chemical modification and extended for the detection of various charged species. Furthermore, the proposed sensor can be integrated with flexible electrode substrate and smartphone for real-time and portable analysis, showing great potential applications in various fields.

## Data Availability

The original contributions presented in the study are included in the article/supplementary material, further inquiries can be directed to the corresponding author.
